# A Mechanism Misregulating p27 in Tumors Discovered in a Functional Genomic Screen

**DOI:** 10.1371/journal.pgen.0030219

**Published:** 2007-12-07

**Authors:** Carrie M Garrett-Engele, Michael A Tasch, Harry C Hwang, Matthew L Fero, Roger M Perlmutter, Bruce E Clurman, James M Roberts

**Affiliations:** 1 Division of Basic Sciences, Fred Hutchinson Cancer Research Center, Seattle, Washington, United States of America; 2 Division of Clinical Research, Fred Hutchinson Cancer Research Center, Seattle, Washington, United States of America; 3 Division of Human Biology, Fred Hutchinson Cancer Research Center, Seattle, Washington, United States of America; 4 Department of Immunology, University of Washington, Seattle, Washington, United States of America; 5 Department of Biochemistry, University of Washington, Seattle, Washington, United States of America; 6 Department of Medicine, University of Washington, Seattle, Washington, United States of America; 7 Howard Hughes Medical Institute, University of Washington, Seattle, Washington, United States of America; The Jackson Laboratory, United States of America

## Abstract

The cyclin-dependent kinase inhibitor p27^KIP1^ is a tumor suppressor gene in mice, and loss of p27 protein is a negative prognostic indicator in human cancers. Unlike other tumor suppressors, the *p27* gene is rarely mutated in tumors. Therefore misregulation of p27, rather than loss of the gene, is responsible for tumor-associated decreases in p27 protein levels. We performed a functional genomic screen in *p27^**+/−**^* mice to identify genes that regulate p27 during lymphomagenesis. This study demonstrated that decreased p27 expression in tumors resulted from altered transcription of the *p27* gene, and the retroviral tagging strategy enabled us to pinpoint relevant transcription factors. *inhibitor of DNA binding 3 (Id3)* was isolated and validated as a transcriptional repressor of p27. We further demonstrated that p27 was a downstream target of Id3 in src-family kinase Lck-driven thymic lymphomagenesis and that p27 was an essential regulator of Lck-dependent thymic maturation during normal T-cell development. Thus, we have identified and characterized transcriptional repression of p27 by Id3 as a new mechanism decreasing p27 protein in tumors.

## Author Summary

Many human cancers express abnormally low amounts of the p27 protein, and this is associated with aggressive tumor behavior and a poor clinical outcome. Surprisingly, the *p27* gene is rarely mutated in these tumors and retains the potential to produce normal amounts of p27 protein. Therefore, understanding the pathways that cause the decrease of p27 protein in cancer cells may lead to the development of new therapies that restore *p27* gene expression to normal levels. We undertook a survey of the mouse genome to identify genes that modulate p27 protein levels in lymphomas. Our analysis discovered *inhibitor of DNA binding 3 (Id3)* as a negative regulator of *p27* gene expression. Additionally, we demonstrated that the *p27* gene is controlled by Id3 during normal embryological development of the thymus. Our results underscore the fact that cancer cells frequently exploit normal developmental pathways as they evolve into increasingly aggressive transformed states.

## Introduction

p27^KIP1^ binds to and thereby prevents cyclin-CDK complexes from phosphorylating their protein substrates [1]. The biological consequence of this molecular interaction in cultured cells is cell cycle arrest, primarily in the G1 phase. Analysis of *p27* ^−*/*−^ mice provides further evidence that p27 is an important regulator of cell proliferation in vivo. *p27* null mice exhibit gigantism and contain proportionally larger, hypercellular organs compared to wild-type siblings [2]. The *p27*-null thymus was disproportionately enlarged compared to the whole animal, though developing thymocyte subsets were maintained in normal proportions. Increased T-lineage proliferation was also seen in the spleen [3]. Further analysis demonstrated that p27 controls cytokine stimulated T-cell proliferation [4,5]. In addition to the cell proliferation phenotype, *p27* ^−*/*−^ mice develop spontaneous pituitary adenomas.

Analyses of tumor susceptibility demonstrated that the *p27* gene is a dose-dependent tumor suppressor gene [6]. That is, a 50% reduction in p27 protein levels was sufficient to predispose *p27^+/−^* mice to tumors in multiple organs, especially following the administration of exogenous carcinogens, or when genetically combined with various oncogenes or deletions of tumor suppressors. Thus, loss of *p27* accelerated the rate of tumor development in *p27* ^−*/*−^ *; Rb^+/−^, p27;pten*, and *p27* ^−*/*−^;Apc^Min+/*−*^ mice [7–9]. In different tumor models, *p27* deficiency increased both the number of tumors and their rate of progression to more aggressive cellular phenotypes [10].

In humans, decreased expression of p27 protein is a negative prognostic indicator in breast, colon, prostate, lung, esophageal, and gastric cancer [11]. Additionally, loss of p27 expression is one of the most clinically significant negative prognostic markers in human breast cancers [11], and its prognostic value improves when combined with other markers [12]. Although reduced p27 protein levels are observed in tumors, both alleles of the *p27* gene are rarely mutated [11]. Therefore, the quantity of p27 protein appears to mediate tumor susceptibility, and misregulation of p27 expression, rather than loss of its gene, is responsible for decreases in p27 protein levels. For this reason, if the pathways that cause p27 misregulation could be inhibited, then the tumor suppressor function of p27 could potentially be restored in cancer cells.

Although the regulation of p27 in normal cells occurs at the transcriptional, translational, and post-translational levels [13–18], most attention in cancer cells has focused on p27 misregulation via ubiquitin-dependent protein degradation, specifically by the SCF-Skp2 E3 ubiquitin-protein ligase [19]. Recently, we analyzed tumorigenesis in knock-in mice expressing a mutant p27 protein (p27T187A) that cannot be ubiquitinated by the SCF-Skp2 pathway. The p27T187A protein was down-regulated in lung tumors induced by an activated *K-ras* allele to the same extent as wild-type p27 protein, and moreover the *p27^T187A^* mice had the same rate of tumor-dependent death as *p27* wild-type mice [20]. Additionally, we observed a substantial decrease in *p27* mRNA in these lung tumors. These data imply that mechanisms other than SCF-Skp2-mediated protein degradation play significant roles in misregulating p27 during tumorigenesis.

To elucidate the molecular mechanisms that contribute to p27 misregulation during lymphomagenesis, we used a functional genomics assay to identify genes that repress p27. We discovered *inhibitor of DNA binding 3 (Id3)* as a candidate negative regulator of p27 in our screen. Id3 is an HLH protein that lacks a DNA binding domain and antagonizes transcriptional activators by stably forming heterodimers unable to bind DNA sequences within target gene promoters and thus down regulates gene expression [21]. We demonstrated that Id3 decreased *p27* transcription during lymphomagenesis and further established that Id3 was part of the pathway by which the src-family protein tyrosine kinase (PTK) p56^lck^ repressed *p27* expression to stimulate proliferation of T-cell progenitors in the thymus.

## Results

### Functional Genomic Assay of Lymphomas from *p27^+/−^* Mice

**Figure 1:**
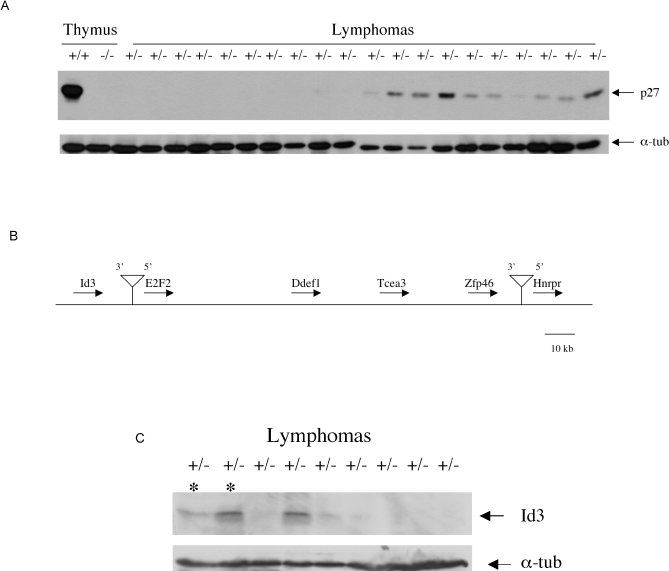
Analysis of M-MuLV Induced Lymphomas from *p27* ^+/−^ Mice (A) Western analysis of p27 protein levels in *p27* wild-type and *p27* ^−*/*−^ thymus tissues and 20 of the 44 *p27* ^+/−^ lymphomas. The blots were probed with α-tubulin as a loading control. (B) A schematic of the region on Chromosome 4 flanking the retroviral insertion sites is shown. The relative positions of *Id3*, *E2F2*, *Ddefl1*, *Tcea3*, *Zfp46*, and *Hnrpr* are indicated. (C) Id3 protein levels in a subset of the lymphomas from (A) *p27* ^+/−^ lymphomas. α-tubulin is shown as a loading control. The tumors with Id3 retroviral insertions near Id3 are indicated with an asterisk. The lymphoma with M-MuLv insertion closest to Id3 has the highest level of Id3 protein. doi:10.1371/journal.pgen.0030219.g001

We used retroviral insertional mutagenesis to screen the mouse genome for genes that modulate p27 protein levels during tumorigenesis. Moloney murine leukemia virus (M-MuLv) integrates throughout the genome and primarily up-regulates nearby genes [22], which induces tumors in vivo and tags the relevant oncogenes by their physical proximity to the retrovirus. Wild-type, *p27* ^−*/*−^, and *p27^+/^* ^−^ heterozygous mice were infected with M-MuLv, which is tropic for the thymus and therefore predominantly induced T-cell lymphomas. Previously, it was demonstrated that after exposure to M-MuLv the *p27^+/−^* heterozygous mice developed lymphomas at an intermediate rate compared to the wild-type and knockout mice [23]. Therefore, given that the *p27* ^+/−^ heterozygotes contain 50% as much p27 protein as wild-type mice [6], this genotype may represent a sensitized background for revealing M-MuLv-induced changes in gene expression that caused further reductions of p27 protein. Our strategy was to identify the subset of tumors arising in *p27* ^+/−^ heterozygotes that contained very low or undetectable quantities of p27 potentially resulting from insertional activation of genes that down-regulated p27. Western analysis of 44 lymphomas induced by M-MuLv in *p27* ^+/−^ mice showed that all of them expressed lower amounts of p27 protein than did normal *p27* ^+/−^ thymus. Indeed, 10/44 tumors had almost no detectable p27 protein (Figure 1A). The uniformly lower amounts of p27 protein in lymphomas compared to normal thymus may be a normal consequence of the increased proliferation characteristic of many tumors. However, we hypothesized that the ten lymphomas with undetectable amounts of p27 protein had suffered an M-MuLv-mediated event that caused a further, pathological reduction in p27 expression. From these ten tumors, retroviral junction fragments were cloned by inverse PCR and verified by DNA sequencing as legitimate M-MuLv-genomic DNA junction fragments (see Materials and Methods). From these we identified 55 unique insertional junction fragments (Table 1), which was in good agreement with the 53 retroviral insertions we had estimated on the basis of Southern data from the tumors (unpublished data).

**Table 1:**
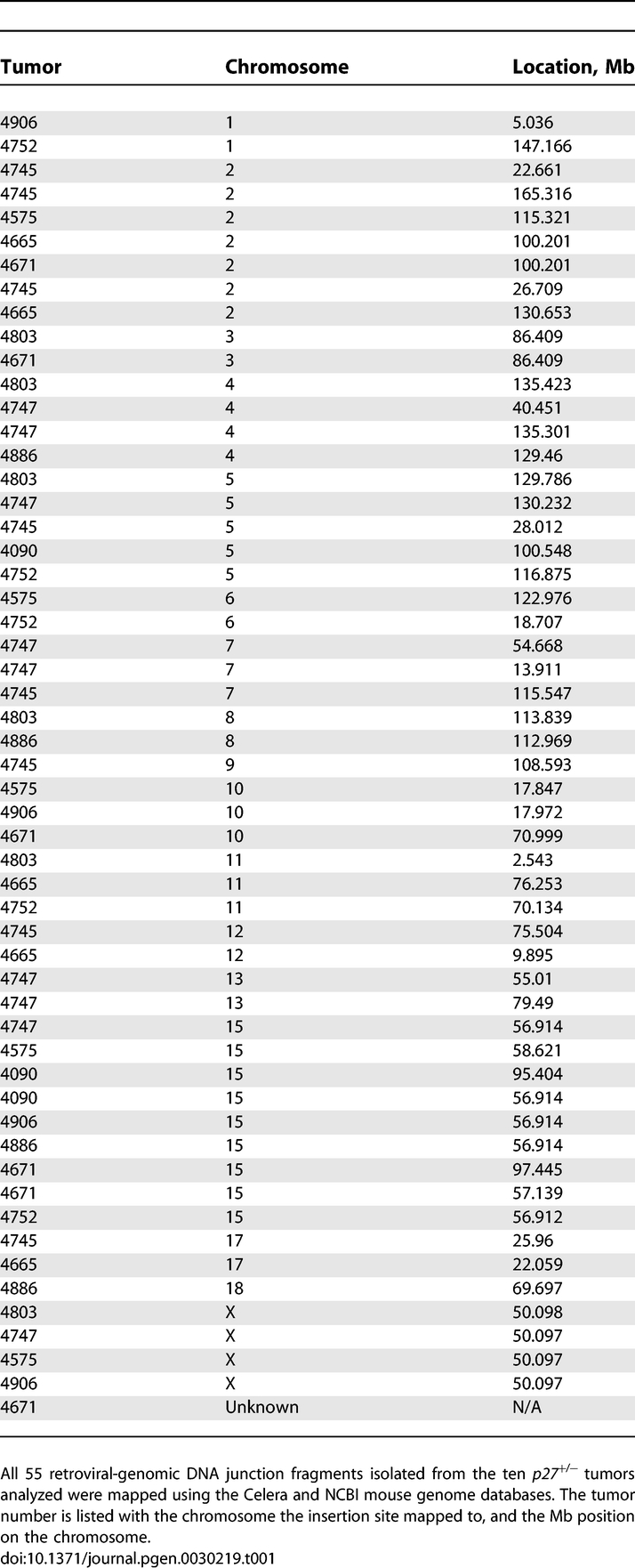
Retroviral Insertion Sites Identified in the *p27* ^+/−^ Lymphomas

DNA sequences of the 55 cloned junction fragments were analyzed using the National Center for Biotechnology Information and the Celera mouse databases. Insertion sites from at least two independent tumors that colocalized to within a 125-kb genomic interval were designated as common insertion sites (CIS). Our analysis revealed seven CIS (Table 2). The gene tagged most frequently in the *p27* ^+/−^ lymphomas was *myc* (seven out of ten). Also, the *C14* CIS [23] was found in four of the ten tumors analyzed; *myb, RORC, cux-1, CIS4,* and *LDLR/Mc7* were each detected in two independent tumors. In a large-scale retroviral insertional mutagenesis to identify cancer genes, 62% of the CIS were found in only two tumors [24]. Similarly, our data indicate 71% of the CIS in *p27* ^+/−^ mice were detected in two tumors. In addition to the above CIS, two CIS defined by insertions in or near different members of a gene family were detected. *Eya1* and *Eya3*, and *Wnt10b* and *Wnt16* were each identified in this way. We categorized these as CIS because, although they were only found near each gene a single time, they involved functionally related members of a gene family. Single insertions in genes within signaling cascades have been detected in other screens, including the Wnt signaling pathway [24]. Finally, single insertions in known CISs were identified for *Jdp2, pim-1, Gfi, rasgrp1*, and *Notch1* (Table 2).

**Table 2:**
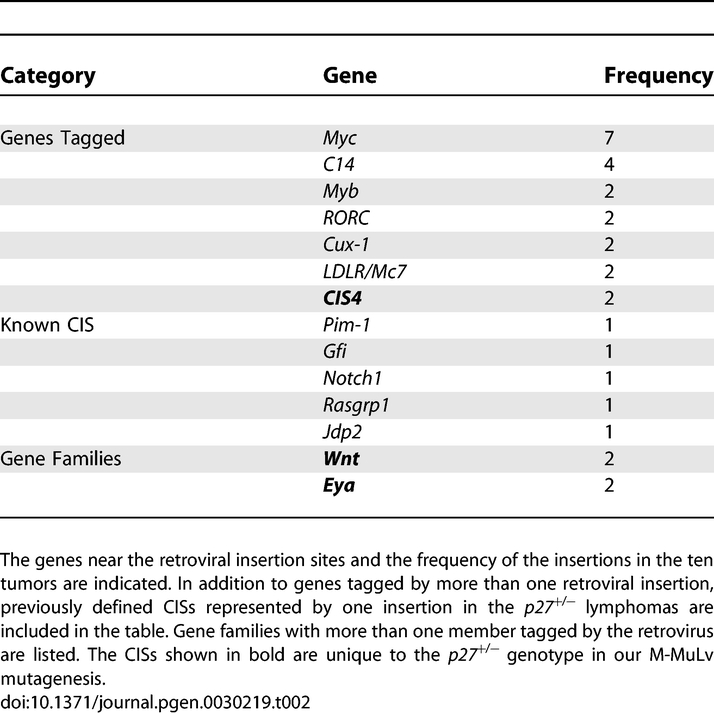
Common Integration Sites in *p27* ^+/−^ Lymphomas

The key question in our approach was how to sort among the many retrovirally tagged genes for the candidates that were most likely to participate in the regulation of p27. We postulated that three types of genes would be tagged in our screen: genes that modulated p27 levels; genes that were oncogenic by mechanisms other than down-regulation of p27; and genes that represented random, unselected sites of retroviral integration. We tentatively assigned a tagged gene to the first, p27-regulatory category if it: (1) was tagged in multiple independent tumors with low levels of p27 protein expression and was therefore unlikely to represent a random, unselected site of integration, and (2) it was not tagged in any tumor arising in *p27* ^−*/*−^ mice. Activation of a gene that down-regulated p27 should provide no selective advantage to cells already lacking the p27 protein. We compared the *p27* ^+/−^ CIS in Table 2 to the CIS identified from 277 junction fragments that were isolated in a similar manner from *p27* ^−*/*−^ lymphomas [23]. Only CIS4 fulfills our criteria for a regulator of p27 because it was defined by insertions from two independent lymphomas (Figure 1B), and insertions at CIS4 were not found in lymphomas from *p27* ^−*/*−^mice (*p* < 0.03). In addition to CIS4, the CIS representing the *Eyes Absent (Eya)* family and the CIS representing the *Wnt* family were not isolated from *p27* ^−*/*−^ mice. Therefore, while the *Eya* and *Wnt* genes may also represent candidate negative regulators of p27, we focused here on CIS4.

### Id3 Is a Target of M-MuLv Activation in CIS4

CIS4 was previously isolated in five retroviral insertional mutagenesis screens in *p27* wild-type mice and designated Evi62 (Retrovirus Tagged Cancer Gene Database). Evi62 insertions were mapped near the *Id3* and *E2F2* genes, but the target gene of the retroviral insertions in Evi62 had not been established. In addition to the *Id3* and *E2F2* genes, the *Ddefl1*, *Tcea3*, *Zfp46*, and *Hnrpr* genes mapped to the region (Figure 1B). Since *Id3* and *E2F2* regulate the cell cycle, we focused on these two as candidate genes. We used western analysis to determine if *Id3* or *E2F2* was up-regulated by the retroviral insertions in our tumors. Analysis of Id3 protein levels in the lymphomas detected high Id3 expression in the tumor with the retroviral insertion immediately adjacent to the *Id3* gene, and moderate Id3 expression in the other, more distantly tagged tumor (Figure 1C). In total, four of the ten lymphomas screened for CIS had high to moderate Id3 expression (unpublished data). One of the tumors with elevated Id3 protein had a retroviral insertion adjacent to the *Notch* gene, which is a known activator of Id3 [25]. The mechanism up-regulating Id3 in the fourth lymphoma was not investigated further. In the other set of 34 lymphomas, with higher expression of p27, 30% had low expression Id3, and no Id3 expression was detectable in the others (unpublished data). No increase in E2F2 protein was observed in any of our tumors with the M-MuLV insertions (unpublished data). Additionally, no effect on p27 transcription by E2F2 was detected by DNA transcription array experiments [26–28]. Therefore, our data suggest Id3 was the likely target of the retroviral insertion at Evi62. In addition to Id3, other genes in the region may also be activated by the retroviral insertions and could potentially regulate p27 in coordination with Id3; however, we analyzed Id3 alone as a negative regulator of p27. In the experiments below, we tested whether there was a causal relationship between Id3 up-regulation and p27 down-regulation.

**Figure 2:**
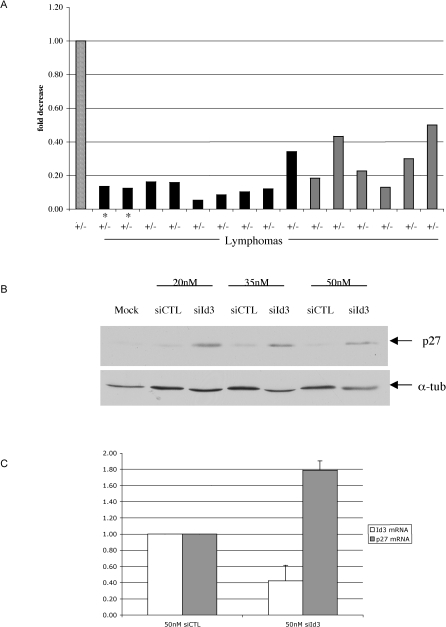
p27 Transcript Is Regulated in *p27* ^+/−^ Lymphomas and Normal Cells (A) *p27* transcript levels in *p27* ^+/−^ thymus and *p27* ^+/−^ lymphomas were determined by quantitative RT-PCR using probe and primer sequences at the border of exons 1 and 2. The amount of RNA in each sample was normalized to *HPRT1* gene expression. The fold change observed is relative to the *p27* transcript levels in the *p27* ^+/−^ normal thymus indicated by the striped bar. The p27 low lymphomas from Figure 1A are shown in black, and lymphomas with higher p27 protein are shown in gray. An asterisk marks lymphomas with retroviral insertions near Id3. (B) p27 protein levels increase dependent on the presence of the Id3 siRNA. α-tubulin expression is shown as a loading control. (C) Quantitative RT- PCR indicates Id3 siRNA decreased *Id3* mRNA and increased *p27* mRNA abundance. *HPRT1* mRNA was used to normalize RNA amounts in each sample. All primer-probe sets are located at exon borders. doi:10.1371/journal.pgen.0030219.g002

Since Id3 regulates transcription, we measured the p27 transcript levels in the lymphomas. Quantitative real time (RT) PCR data revealed that *p27* mRNA was reduced in all of the *p27* ^+/−^ lymphomas relative to normal *p27* ^+/−^ thymus, with a significantly greater reduction in *p27* mRNA abundance in the set of lymphomas expressing very low or absent p27 protein (Figure 2A). Therefore, control of mRNA expression appeared to be a general mechanism for decreasing p27 protein levels in these lymphomas. To determine whether Id3 regulates *p27* transcript levels, we knocked down *Id3* expression in NIH3T3 cells using *Id3* siRNAs. Three concentrations of pooled *Id3* siRNAs were transfected into cells, and all siRNA concentrations increased p27 protein levels compared to cells transfected with a nonspecific control siRNA (Figure 2B). Quantitative RT-PCR on RNA isolated from these cells confirmed that *Id3* mRNA decreased and that *p27* mRNA increased, dependent on the presence of the *Id3* siRNA (Figure 2C). Thus, Id3 repressed *p27* transcript levels in NIH 3T3 cells.

### Id3 Represses *p27* Gene Expression

**Figure 3:**
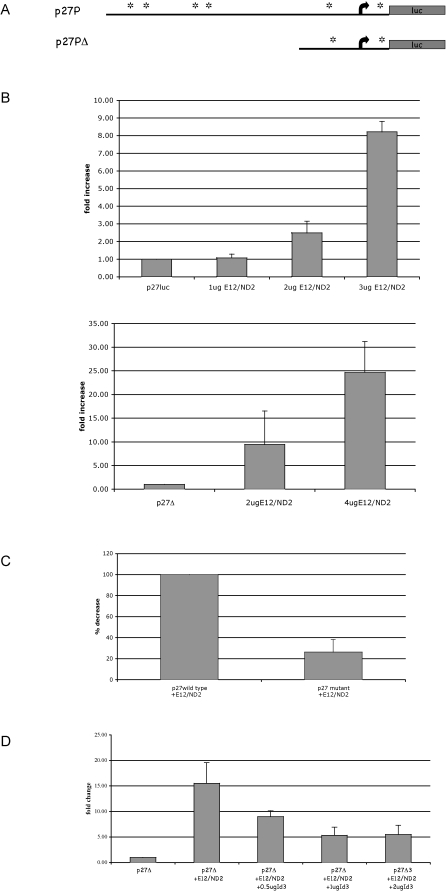
Id3 Regulates Expression of p27 Promoter Reporter Construct (A) A schematic of the full-length *p27* promoter and a minimal *p27* promoter are shown. The E-box sequences CANNTG are marked by asterisks. (B) The E12/NeuroD2 E-protein heterodimers activate the full-length and minimal *p27* promoters. (C) The percent induction of the mutant *p27* minimal promoter, with three of the four conserved bases in the E boxes mutated (CANNTG to CGNNAT), is shown relative to the wild-type promoter. (D) Id3 represses E-protein activation of the *p27* minimal promoter. The SV40-β-galactosidase or CMV-β-galactosidase plasmids were used as an internal control for each sample. doi:10.1371/journal.pgen.0030219.g003

The HLH protein Id3 represses transcription by interacting with transcription factors to form heterodimers unable to bind DNA. Previous experiments demonstrated that coexpression of the bHLH proteins E12 and NeuroD2 led to increased p27 protein in cells [29]. However, these experiments did not address whether this increased p27 expression was a direct or indirect effect of the bHLH proteins, nor whether it occurred at the level of p27 transcription. The 2.0-kb mouse *p27* promoter contains six potential E-box sequences (CANNTG). We tested the ability of bHLH heterodimers to stimulate transcription from a full-length *p27* promoter-luciferase fusion gene and a minimal promoter containing two of the six E-box sequences (Figure 3A). While neither E12 nor NeuroD2 alone affected *p2*7 promoter activity (unpublished data) as expected [30], cotransfection of increasing amounts of E12/NeuroD2 caused a dose-dependent increase in both the full length and minimal *p27* promoter activity (Figure 3B). Expression of the *p2*7 promoter-luciferase fusion gene constructs was also stimulated by E12/E47 heterodimers, which are bHLH proteins expressed in lymphocytes (unpublished data). This activation was dependent on the presence of the two E boxes (CANNTG) and one imperfect E box (GACCTG) in the *p27* minimal promoter, as the *p27* minimal promoter with these sites mutated to CGNNAT and GGCCAT respectively could only be induced to 26% of the wild-type promoter (Figure 3C). This residual induction was likely due to fortuitous E-box sequences in the expression vector itself [31]. Furthermore, titration of Id3 abrogated the stimulatory effect of E12/ND2 on the *p27* minimal promoter (Figure 3D). The effect of Id3 was specifically to antagonize bHLH protein-stimulated transcription, because in the absence of cotransfected bHLH proteins, Id3 had almost no detectable effect on basal, low-level *p27* promoter expression (unpublished data). The results of these experiments suggest that bHLH proteins directly activate the *p27* promoter and that Id3 represses *p27* transcription by inhibiting bHLH protein function.

**Figure 4:**
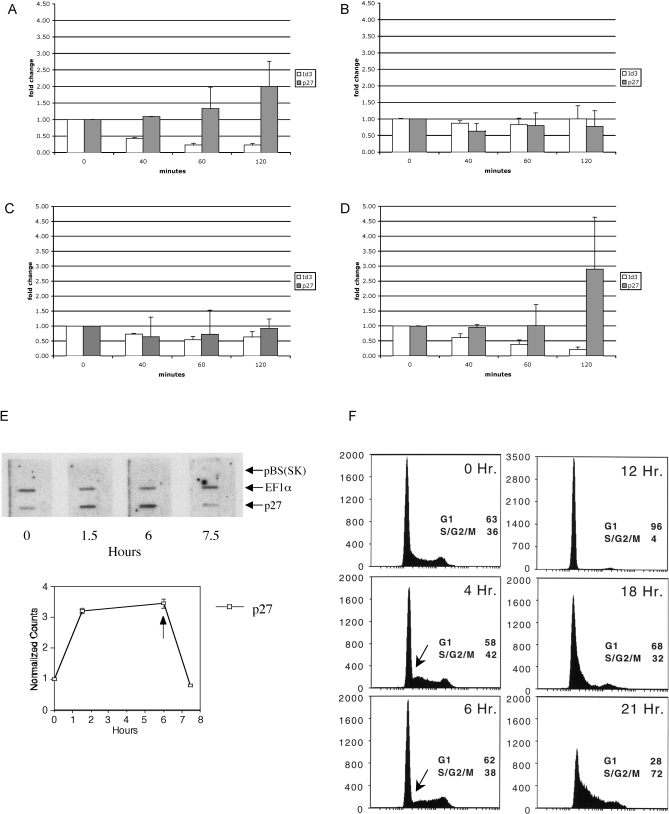
p56^Lck^ Regulates *p27* Gene Expression Via Id3 in Lymphoma Cells Quantitative RT-PCR detected transcript levels for *Id3* and *p27* in PP1 inhibited (A) Lck-expressing cells LGY-6871, (B) SV40–180 large T-protein transformed cells, (C) LGY-6871 cells infected with an Id3 retrovirus, and (D) LGY-6871 cells infected with the control retrovirus are shown in the graph. PP1 was added immediately after the time-0 timepoint was collected, and later aliquots were collected at the specified times. The Id3 probe and primers detect endogenous and retroviral expressed *Id3*. *HPRT1* gene expression was used to normalize each sample. The fold change in transcript levels is relative to the 0 timepoint. (E) Representative autoradiograph of nuclear run-on assays from PP1 inhibited Lck-expressing cells LGY-10442–2 is shown and quantitative data from three experiments. The *p27* transcript levels were normalized to *EF1α* transcript levels. PP1 was added to the culture after the 0 timepoint and washed out after 6 h as indicated by the arrow. (F) FACS analysis of PP1-inhibited Lck-transformed lymphoma cells LGY-10442–2. The cells were treated as described for (E). The arrow indicates depletion of early S-phase cells doi:10.1371/journal.pgen.0030219.g004

To further examine Id3 regulation of *p27* in cancer cells, we studied lymphoma cell lines in which the src-family protein tyrosine kinase p56^lck^ could be conditionally regulated. p56^lck^ activity increased *Id3* transcript levels in cultured lymphocytes [32], and cell lines derived from lymphomas arising in mice overexpressing p56^lck^ [33] provided a system to assay Id3-mediated repression of *p27*. Cells were treated with a pharmacologic inhibitor highly specific for src-family protein tyrosine kinases, PP1 [34]. Analysis of *Id3* mRNA abundance by quantitative RT-PCR confirmed that this transcript rapidly declined 5-fold within 1 h following inhibition of p56^lck^ activity. After *Id3* expression declined and Id3 protein disappears because of its short half-life [35], *p27* mRNA increased an average of 2-fold by 2 h (Figure 4A). A cell line derived from thymic lymphomas arising in SV40 large T-protein transformed mice was used as a control for PP1 specificity for p56^lck^ [36]. As expected if p56^lck^ is the kinase required for modulating Id3 and p27 transcript abundance, *Id3* and *p27* mRNA levels remained essentially unchanged after addition of PP1 to these cells (Figure 4B), consistent with the transformed phenotype of these cells being independent of Lck activity. Thus, p56^lck^ activity was required for maintaining *Id3* mRNA expression and repressing *p27* transcript levels.

We further showed that enforced *Id3* expression rescued *p27* repression in lymphoma cells in cells treated with the pharmacologic inhibitor of p56^lck^. Lck-transformed cells infected with an *Id3*-expressing retrovirus or control retrovirus were treated with PP1 and assayed for *Id3* and *p27* transcript abundance. Cells transduced with the *Id3* expression vector continued to express the *Id3* mRNA at close to physiological levels (>50%) after addition of PP1, and the *p27* mRNA remained repressed (Figure 4C). The results from the control retrovirus infected cells were indistinguishable from the uninfected cells; the *Id3* transcript was decreased 5-fold, and the *p27* transcript was increased almost 3-fold after addition of PP1 (Figure 4A and 4D). Since Id3 acts as transcription repressor, the *p27* transcription rate was examined using nuclear run-on assays. A rapid 3-fold induction of *p27* transcription was observed following p56^lck^ inhibition and decreased *Id3* expression, and the converse transcriptional silencing of the *p27* gene soon after inhibitor removal and reactivation of p56^lck^ (Figure 4E). These results demonstrate that Id3 was sufficient to repress accumulation of the *p27* mRNA via a reduced rate of *p27* transcription.

The increased *p27* transcription that follows p56^lck^ inhibition delayed progression through the cell cycle. Initially, Lck inhibition resulted in depletion of early S-phase cells, followed over time by an accumulation of cells with a 2N (G1) content of DNA (Figure 4F). Removal of PP1 resulted in a near-synchronous progression of the culture into S phase (Figure 4F). Western-blot analysis of lysates from PP1-treated cells showed a rapid accumulation of p27, its association with cyclin E-containing complexes, and a loss of in vitro histone kinase activity in immunoprecipitates containing cyclin E (unpublished data). Conversely, following washout of PP1, *p27* mRNA levels rapidly fell to baseline though the protein remained elevated for an additional 12 h falling coincident with induction of cyclin E-associated kinase activity and S-phase entry (unpublished data). Therefore, the increased transcription of *p27* due to loss of Lck and Id3 activity resulted in a reversible cell cycle arrest in lymphoma cells.

### p56^lck^ and Id3 Regulate *p27* during Thymocyte Maturation

To investigate the repression of p27 by Id3 and p56^lck^ in vivo, we tested whether a genetic interaction could be detected during normal thymic development in mice. Thymic maturation is punctuated by differential induction and silencing of CD4 and CD8 expression. The most immature lymphoid cells are CD4^−^CD8^−^ double-negative (DN) cells [37]. Within the DN compartment, expression of CD25 and CD44 further defines four subsets (DN1 through DN4) of increasing maturity. The DN4 subset is the immediate antecedent to the double-positive (DP) population [38]. Mice deficient in p56^lck^ have impaired DN3 to DN4 maturation and consequent constriction of the DP compartment, resulting in hypocellular thymi proportionately enriched in DN cells [39]. Similarly, overexpression of *p27* also inhibits the DN3 to DN4 transition in a dose-dependent fashion [40]. Therefore, as observed in lymphoma cells Lck activity may repress *p27* gene expression during thymocyte maturation.

**Figure 5:**
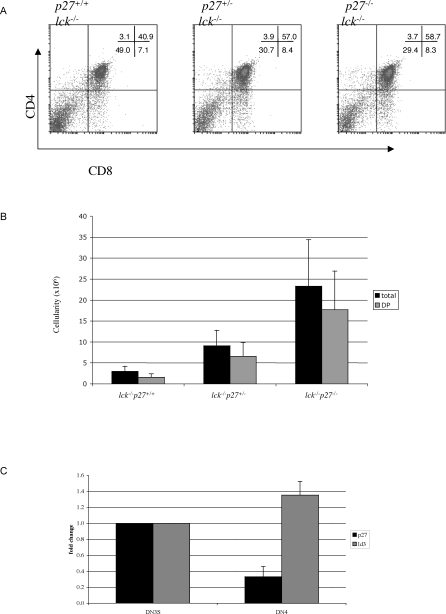
Loss of *p27* Rescues the *lck* ^−*/*−^ Phenotype (A) Representative FACS analysis of thymi from *lck* ^−*/*−^ *p27^+/+^*, *lck* ^−*/*−^ *p27* ^+/−^, and *lck* ^−*/*−^ *p27* ^−*/*−^ mice indicating loss of one copy of the *p27* gene rescues the *lck* ^−*/*−^ phenotype. (B) The total cellularity for thymi and DP cells from all three genotypes are shown in the graph. Data were collected from three *lck* ^−*/*−^ *p27^+/+^*, seven *lck* ^−*/*−^ *p27* ^+/−^, and seven *lck* ^−*/*−^ *p27* ^−*/*−^ mice. (C) Quantitative RT-PCR detected transcript levels for *Id3* and *p27* in wild-type small DN3 and DN4 cells. The fold change in the mRNA levels in the DN4 cells is relative to the DN3 cells. doi:10.1371/journal.pgen.0030219.g005

As a test of whether p27 is downstream of Lck during thymocyte maturation, *p27* ^−*/*−^ mice were crossed to *lck* ^−*/*−^ mice, and the phenotypes of the progeny were analyzed for rescue of the proliferative and developmental arrest caused by the loss of Lck activity. The *lck* ^−*/*−^ *p27* ^+/−^ and *lck* ^−*/*−^ *p27* ^−*/*−^ animals displayed a proportionate and absolute expansion of the DP compartment (Figure 5A), consistent with an increase in either the number of cells maturing or proliferating, or both. Also, total thymus cellularity progressively increased in inverse relationship to *p27* gene dosage, although it did not reach wild-type numbers (Figure 5B). This reflected a numeric expansion of both the DN and DP compartments, with proportionately greater increase in the latter. Additionally, the DN compartment in the *lck* ^−*/*−^ *p27* ^−*/*−^ mice appeared enriched in the DN4 subset, indicative of a reduced threshold for DN3 to DN4 progression (unpublished data). Thus loss of *p27* partially rescued the defects in *lck* ^−*/*−^ mice. If Lck repressed p27 through Id3, we would expect increased levels of Id3 at the time *p27* transcript is reduced. We compared *p27* and *Id3* transcript levels in small DN3 cells and DN4 cells from wild type mice. As cells transitioned to the DN4 stage *p27* mRNA decreased 3-fold compared to the DN3 stage, and *Id3* mRNA increased 1.3-fold (Figure 5C). Therefore, as observed in the lymphoma cells, there was a correlation between decreasing *p27* transcript levels and increasing *Id3* transcript levels, suggesting that Id3 repressed *p27* gene expression at the DN3 to DN4 transition. These results extend our molecular observations connecting Lck and p27 through the action of Id3 in lymphoma cells to normal thymocyte development.

## Discussion

We describe a functional genomics assay employing M-MuLv insertional mutagenesis screen to detect negative regulators of a tumor suppressor gene. We analyzed lymphomas from *p27* ^+/−^ mice and discovered Id3 as a repressor of p27 in cancer cells. Since the *Id3* CIS was detected in *p27* ^+/−^ tumors with low p27 protein, we propose Id3 contributed to lymphomagenesis by causing the observed decrease in p27 protein. Consistent with Id3 functioning as a transcriptional repressor, *p27* mRNA amounts were greatly reduced in the tumors with *Id3* tagged by the retrovirus. Although decreases in *p27* transcription in tumors or cancer cell lines have not been widely reported, the predisposition of *p27* ^+/−^ mice to tumors demonstrates a 50% reduction in *p27* mRNA levels is physiologically relevant. Moreover, we previously observed significantly reduced *p27* transcript in a murine lung tumor model and in a subset of human breast cancers [20]. Therefore, decreased *p27* mRNA abundance may occur in multiple types of cancer, and our isolation of Id3 as a negative regulator of *p27* implies a novel mode of regulation for p27 in lymphomas.

In agreement with transcriptional regulation of *p27* decreasing p27 protein in tumors, all of the *p27* ^+/−^ lymphomas with low p27 protein analyzed for CIS had reduced levels of *p27* mRNA. In addition to *Id3*, we identified single specific insertions in *p27* ^+/−^ mice near *Wnt10b* and *16* as well as *Eya1* and *3*. Cofactors of Eya proteins and Wnt1 repress *p27* transcription [41,42]. In total, among the ten lymphomas in which we were able to document very low p27 protein and mRNA expression, seven had either up-regulated Id3 protein or retroviral insertions near the *Wnt* or *Eya* genes. Two of the remaining lymphomas had insertions in the *Myb* locus. The *p27* promoter contains a Myb binding site [43], and Myb collaborates with Hes1, a known negative regulator of p27 [44]. However, the *Myb* CIS was also found in lymphomas arising in *p27* ^−*/*−^ mice. Thus, if Myb is a negative regulator of p27 it is also likely to have p27-independent effects on tumorigenesis. Therefore, transcription factors or cofactors were identified in nine out of the ten tumors; however, *Id3* represented the sole CIS not found in *p27* ^−*/*−^ lymphomas.

Id3 and p27 have opposing effects on proliferation and differentiation. Overexpression of *Id1–3* genes increased cell proliferation, and antisense *Id1–3* delayed reentry of arrested cells into the cell cycle [45], whereas increased p27 protein arrests cells in G1. Also, overexpression of *Id1* and *Id2* in the thymus promotes the development of lymphomas [46,47]. In *Id1* ^−*/*−^ *Id3* ^−*/*−^ knockout mice, neuroblasts prematurely withdraw from the cell cycle and p27 protein levels are elevated [48] consistent with our luciferase assay data that Id3 regulates *p27* transcription via interference with neural bHLH proteins. Additionally, in a wound healing model, Id3 repressed ELK1 activation of *p27* gene expression [49]. Furthermore, inverse mRNA expression patterns of *p27* and *Id3* have been observed in cells [50]. Increased Id3 and decreased p27 protein levels were observed when an oncogene was transfected into cells [51]; however, a function for Id3 regulating p27 in tumorigenesis was not investigated. Although regulation of *p27* transcription has been reported by Id3 in wound healing [49] and other transcription factors in cell culture [18], our results offered the first evidence that Id3 directly regulated *p27* transcription in cancer cells, and moreover indicated that, in vivo, it could cause the misregulation of *p27* during tumorigenesis.

The variety of mechanisms controlling transcriptional misregulation of *p27* in human cancers remains to be fully investigated. Id3 is overexpressed in many cancer types [52], and loss of p27 is a significant negative prognostic indicator for many types of human cancers [11]. Furthermore, analysis of human leukemias by DNA microarrays found increased *Id3* gene expression and decreased *p27* gene expression in the samples (Ross_Leukemia and Schmidt_Leukemia, Oncomine database [53]). Therefore, Id3 misregulation of *p27* transcription may be an important mechanism in human cancers.

Our data demonstrating the control of *p27* transcription as a central mode of regulation downstream of Lck suggested p27 may be regulated similarly in normal developmental processes dependent on p56^lck^ signaling, including β-selection [54]. The role of bHLH/Id proteins in lymphoid development is well established [30]. However, the genes regulated by bHLH/Id proteins during β-selection remain unknown. Previously, a cell cycle defect was observed in *E2A* ^−*/*−^ lymphocytes when the bHLH protein E47 was added to these cells, a phenotype that could indicate increased *p27* expression [30]. Other observations also suggest *p27* may be regulated by the bHLH/Id proteins during β-selection. Specifically, that DN4 cells had reduced *p27* expression compared to small, quiescent DN3 cells [55], and impaired DN3 to DN4 maturation was seen with increasing dosage of transgenic p27 expression [40]. Our data confirmed that *p27* transcript was decreased in DN4 cells relative to DN3 cells and correlated the *p27* mRNA decrease with increased *Id3* mRNA at the DN3 to DN4 transition. Therefore, we concluded *p27* may be a critical target of regulation by Lck through Id3 during thymic β-selection.

We further observed that in a gene-dosage fashion, *p27* ^+/−^ and *p27* ^−*/*−^ partly relieved the *lck* ^−*/*−^ phenotype, augmenting the efficiency of DN to DP maturation and consequent thymus cellularity. That the DN compartment expanded in absolute cell numbers was an unexpected observation and may reflect more developmental niches available throughout the thymus, as the thymic epithelium increases in response to both maturation and expansion of the lymphoid compartment [56,57] as well as a consequence of the *p27* mutation [58]. In more preliminary studies, we examined the effect of *p27* mutation on the absolute block in development in a *lck/fyn* double mutant (M. Tasch and R. Perlmutter, unpublished observations). The residual thymic maturation in the *lck* mutant is due to functional redundancy between Lck and a related Src-family kinase, Fyn, and loss of both p56^lck^ and p59^fyn^ results in severe thymic hypocellularity and developmental arrest at the DN3 stage. Here, *p27* ^−*/*−^ relieved the DN3 arrest and allowed accumulation of a DN4 compartment. In parallel, very low level of expression for CD4 and CD8 was observed, consistent with thymocytes making the initial step in the DN to DP transition. However, this maturation was abortive as bona fide DP cells failed to accumulate. These results are consistent with p27 being a critical target of Lck signaling during thymic maturation, but also indicate that Lck has targets in addition to p27 that are essential for full thymic maturation.

In conclusion, our novel retroviral insertional mutagenesis screen discovered a repressor of the tumor suppressor p27, and our subsequent analysis identified transcriptional misregulation of the *p27* gene by Id3 as a new mechanism controlling p27 levels in lymphomas. This study highlights the fact that developmental pathways that regulate gene expression in normal cells are often coopted during tumor cell evolution. Thus, Id3 is an essential downstream target of Lck during normal thymic maturation and is activated to drive p27 down-regulation in Lck-driven thymic lymphomas. Finally, having demonstrated the importance of misregulating p27 at the level of gene transcription during lymphomagenesis, it now becomes crucial to understand which transcriptional pathways cause misregulation of *p27* in human cancers, and how frequently this occurs.

## Materials and Methods

### Mice and M-MuLV mutagenesis.

M-MuLv infection and genotyping of the *p27* ^+/−^ mice was previously described in [23]. Animals were euthanized when they developed signs of morbidity, and the lymphomas were snap frozen in liquid nitrogen at necropsy. For thymus analysis, *p27* ^−*/*−^ mice on the 129 background were mated to *lck* ^−*/*−^ mice maintained on the C57BL/6 background, and the compound heterozygous F1 mice were then backcrossed with *lck* ^−*/*−^ animals. The *lck* ^−*/*−^ *p27* ^+/−^ mice were mated with either *lck* ^−*/*−^ *p27* ^+/−^ or *lck* ^−*/*−^ *p27* ^−*/*−^ mice and the thymi from *lck* ^−*/*−^ *p27^+/+^, lck* ^−*/*−^ *p27* ^+/−^ *,* and *lck* ^−*/*−^ *p27* ^−*/*−^ progeny analyzed. Mice carrying targeted disruptions of the *p27* and *lck* genes, and the procedures and reagents used in genotyping, have been described previously [3,39].

### Western blotting.

Small pieces of normal thymus from wild-type mice and lymphomas from *p27* ^+/−^ mice were homogenized in lysis buffer (1× PBS, 1% NP-40, 0.1% SDS, 0.5% sodium deoxycholate, 1 mM dithiothreitol, 10 mM sodium flouride, 1 mM sodium orthovanadate, 10 μg/ml leupeptin, 10 μg/ml aprotinin, 10 μg/ml pepstatin-A, and 1 mM phenylmethylsulfonylfluoride). NIH 3T3 cells were lysed in the same buffer as above. Samples were sonicated, and protein concentrations were determined using the Bio-Rad protein assay. A total of 40 μg of protein was separated on 12% SDS-PAGE gels, and the proteins were transferred onto polyvinylidene diflouride membranes (Perkin Elmer). The membranes were blocked in 1× PBS, 0.1% Tween-20, and 5% milk. After blocking, the membranes were incubated with primary antibodies diluted 1:1,000 in 1× PBS, 5% milk, and 0.1% Tween-20 overnight at 4 °C. Id3 (C-20) and p27 (C-19) rabbit polyclonal antibodies from Santa Cruz Biotechnology and the α-tubulin (clone DM1A) mouse monoclonal antibody from Sigma were used. ECL (Amersham) was used for immunodetection.

### DNA isolation and inverse PCR.

Genomic DNA was isolated from 44 *p27* ^+/−^ lymphomas and inverse PCR performed as previously described [23]. After two rounds of I-PCR the products were gel purified and cloned using the Topo cloning system (Invitrogen) following manufacturers protocol. Plasmids were sequenced using M13–20 or M13 reverse primers and sequencing reactions were carried out at the FHCRC automated sequencing shared resource. The DNA sequence data were blasted against the NCBI, Celera, and Ensembl mouse databases. The statistical significance of the CIS4 retroviral insertions in the *p27* ^+/−^ versus the absence in the *p27* ^−*/*−^ lymphomas was determined using Fisher's exact test at http://www.exactoid.com/fisher/index.php.

### Tissue culture and retroviral infections.

HEK 293T and Phoenix ecotropic cells were grown in Dulbecco's modified Eagle medium (DMEM) containing 10% bovine growth serum (Hyclone), 1mM sodium pyruvate, 2 mM L-glutamine, and 2 μg/ml penicillin-streptomycin (Life Technologies). NIH 3T3 cells were grown in same medium as above with the exception of 10% fetal bovine serum (Hyclone). Thymic lymphoma cell lines LGY-6871, LGY-10442–2, and SV40–180 were maintained in RPMI 1640 supplemented with 10% fetal bovine serum, 2 mM L-glutamine, 0.1 mM nonessential amino acids, 50 U/ml penicillin G, and 50 μg/ml streptomycin, 2.0 mM HEPES buffer (pH 7.4), and 100 μM β-mercaptoethanol. All cell lines were maintained at 37 °C in 5% CO_2_.

For retroviral infections, the mouse Id3 cDNA (provided by B. Christy) was cloned into the pQCXIP vector (BD-Clontech). Ecotropic Phoenix cells were transfected with 15 μg pQCXIP-Id3 or pQCXIP using Fugene 6 (Roche). After 24 h the medium was changed to cRPMI, and the cells were moved to 34 °C. The LGY-6871 cells were plated in a 10-cm dish and allowed to recover 4 h before infection. The viral supernatant was collected 48 and 72 h after transfection and passed through a 0.22-μm filter onto the LGY-6871 cells and 1 μg polybrene added. The infections were done at 34 °C. Twenty-four hours after the last infection the cells were centrifuged at 1,000 rpm for 5 min, and the pellets resuspended in cRPMI medium and moved to 37 °C. Selection with 0.5 μg/ml puromycin (Calbiochem) began 48 h after infection for 4 d. Western analysis confirmed the expression of Id3.

### RNA isolation and RT-PCR.

RNA was extracted from pieces of frozen lymphomas, normal thymi, normal thymocytes, NIH3T3 cells, or thymic lymphoma cells LGY-6871 and SV40–180 following the Trizol protocol (Invitrogen). cDNAs were generated by reverse transcribing 1 μg of total RNA using oligo dT and the Taqman reverse transcription kit (Applied Biosystems). The cDNAs were diluted 1:10, and 5 μl added to each reaction containing Taqman master mix at 1× concentration and the p27 Mm00438167_g1, Hprt1 Mm00446968_m1, Id3 Mm00492575_m1, or Id3 Mm01188138_g1 Assay on Demand primers and probe (Applied Biosystems). Each 50-μl reaction was done in triplicate. The Taqman analysis for the PP1-inhibited cells was performed on two to three separate experiments. Taqman RT-PCR reactions were performed using an ABI PRISM 7900HT sequence detector and analyzed by the SDS2.2 software. Probe sequences were as follows:

Mouse p27: AGGAAGCGACCTGCTGCAGAAGATT

Mouse HPRT: AGGTTGCAAGCTTGCTGGTGAAAAG

Mouse Id3: GGCACCTCCCGAACGCAGGTGCTGG

Mouse Id3: CCGATCCAGACAGCTGAGCTCACTC

### siRNA transfections.

NIH 3T3 cells were transfected with 20 nM, 35 nM, or 50 nM pooled Id3 siRNA duplexes or siControl nontargeting siRNA duplexes (Dharmacon) using siLentfect lipid (Bio-Rad) according to the manufacturer's protocol. Cells were harvested for RNA isolation or protein lysates 24 h after transfection.

### Luciferase and **β**-Galactosidase assays.

The mouse p27 promoter constructs were generated by cloning a 2.2-kb BamHI-BspEI fragment or a 550-bp SacI-BspEI fragment into the pGL-2 luciferase vector (Promega). The mouse E12 and mouse NeuroD2 plasmids were provided by S. Tapscott and J. Olson, respectively. The internal control plasmids were SV40-β-galactosidase or CMV-β-galactosidase, and the CMV-luciferase plasmid was used as a positive control. HEK293T cells were plated on 60-mm dishes, transfected using calcium chloride method or Fugene 6(Roche), and harvested 48-h after transfection for assays. Each transfection experiment was repeated three to four times to ensure reproducibility. The luciferase assays using Steady-Glo luciferase substrate (Promega) and β-Galactosidase enzyme assay system (Promega) were used according to manufacturers protocol. An EG&G plate reader was used for all luciferase experiments, and a Labsystems Multiskan Plus plate reader was used for the β-Galactosidase assays. Each luciferase and β-Galactosidase reaction was done in triplicate and the data averaged.

### Site-directed mutagenesis.

E boxes 1 and 2 and the imperfect E box were mutated to CGNNAT and GGNNAT using the QuikChange Multi Site-Directed Mutagenesis kit (Stratagene) following the manufacturer's protocol. Primer sequences were as follows:

Ebox1: GCCCTCCAGTACGCTATATCACTGAAGCCTCGAG

Ebox2: CCTGGCTCTGCTCCGTTATACTGTCTGTGTGCAGTCG

I E box: GCCTCTCTTCCCCAGGCCATCGCGCTACTGCG

### Lck-inhibition.

The src-family–specific tyrosine kinase inhibitor PP1 (Biomol) was used in in vitro cultures at 5 mM, diluted from a 5-mM stock in DMSO directly into LGY-6871, LGY-6871+Id3, LGY-6871+pQCXIP, or SV40–180 culture**s**. Aliquots were removed from cultures at indicated time points, washed in cold PBS, and cell pellets either (1) snap frozen and maintained at −80 °C for later protein extraction, (2) immediately resuspended in Trizol for subsequent RNA extraction, (3) used immediately for DNA staining, or (4) prepped for nuclei isolation.

### DNA content analysis.

LGY experimental and SV40 control cells were washed in cold PBS and resuspended in a solution of 4.0 mM sodium citrate (Sigma), 30 U/ml RNase, 0.1% Triton X-100 (Sigma), and 50 mg/ml propidium iodide (Sigma). Cells were incubated at 37 °C in the dark for 10 min, after which 1/10 volume of 1.38 M sodium chloride was added. Cells were then analyzed using a FACScan flow cytometers (Beckton-Dickenson) and Cell Quest (Beckton-Dickenson) analysis software.

### Transcription run-on analysis.

Intact nuclei from LGY experimental and SV40 control cells were isolated in hypotonic Tris buffer containing 0.25% nonident P-40 (Sigma), and nascent mRNA transcripts were radiolabeled by incubation in buffer containing 60 mM each ATP, CTP, GTP, and 20 ml a^32^P-UTP (4,000 Ci/mmol) [59]. Nuclei were treated with RNase-free DNase and proteinase K, and RNA was isolated by phenol:chloroform extraction and ethanol precipitation. Target DNA was linearized and denatured prior to immobilization on polyvinylidene fluoride (PVDF) membranes according to the manufacturer's instructions (Schleicher and Scheull). Target sequences included pBluescript (SK) (Stratagene), murine p27 cDNA, and murine elongation factor1a (EF1a) cDNA. Hybridization was carried out for 18 h at 65 °C, and subsequently membranes were washed stringently and exposed to autoradiography media and quantitated by phosphorimage analysis.

### Flow cytometry.

Thymus tissue from *lck* ^−*/*−^ *p27^+/+^, lck* ^−*/*−^ *p27* ^+/−^ *,* and *lck* ^−*/*−^ *p27* ^−*/*−^ mice was harvested after euthanasia, and single-cell lymphocyte suspensions prepared by mechanical disruption with scalpel followed by maceration with frosted glass slides. Cell preparations were washed with cold PBS and red blood cells depleted with hypotonic ammonium chloride per published protocols. Antibodies specific for murine CD4, CD8, CD44, and CD25 and conjugated to fluorescein isothiocyanate (FITC), R-phycoerytherin (PE) or biotin, and streptavidin conjugated to PE-Cy5 were obtained from BD/Pharmingen and used at empirically derived concentrations on samples of 1 × 10^6^ cells. Staining buffer consisted of PBS with 5% FCS, 5 mM HEPES buffer (pH 7.4), and 1 mM sodium azide. After staining and extensive washing, cells were fixed in 2% paraformaldehyde and analyzed with a FACScan flow cytometers and Cell Quest software (both Beckton-Dickenson). To isolate DN3 and DN4 cells for mRNA, cells were sorted using a FACS ARIA flow cytometer and FACSDiva software (both products from Beckton Dickinson) on the basis of CD44 and CD25 expression and cell size, as determined by forward light scatter characteristics. Sorted cells were then disrupted and extracted for total RNA as detailed above.

## Supporting Information

### Accession Numbers

Accession numbers for genes mentioned in this paper from the National Center for Biotechnology Information (NCBI) (http://www.ncbi.nlm.nih.gov) are *Id3* (NM_008321), *E2F2* (NM_177733), *Ddefl1* (NM_001008232), *Tcea3* (NM_011542), *Zfp46* (NM_009557), and *Hnrpr* (NM_028871).

## Abbreviations

CIS: common insertion sites
DN: double negative
DP: double positive
*Eya*: *Eyes Absent*
*Id3*: *inhibitor of DNA binding 3*
M-MuLv: Moloney murine leukemia virus
p27T187A: p27 mutant protein
RT: real time
